# Supplementary material for: Deletion of wfs1 Impairs Oligodendrocyte Precursor Cells Dorsal Distribution and Myelination Through the wfs1-hmgcs1 Axis in Zebrafish
Source: Biology (Basel). 2026 Aug 16;15(16):1402. doi: 10.3390/biology15161402 (PMC13510151; doi:10.3390/biology15161402)
Supplement: Supplementary file 1 [file biology-15-01402-s001.zip › File S1-Transcriptome sequencing data and differentially expressed genes (DEGs) in wfs1-- zebrafish at 5 dpf/2.KEGG/wfs1KOvsWT/DOWN/src/dre04330.html]

dre04330


- dre:678649

- Down regulated genes

678649(-1.83750740546989)

- dre:393500

- Down regulated genes

393500(-6.69197144060497)

- dre:140422

- Down regulated genes

140422\_1(-1.39741032138353)

- dre:557340

- Down regulated genes

557340(-1.39758065205256)

Close
